# Diameter-dependent phase selectivity in 1D-confined tungsten phosphides

**DOI:** 10.1038/s41467-024-50323-y

**Published:** 2024-07-13

**Authors:** Gangtae Jin, Christian D. Multunas, James L. Hart, Mehrdad T. Kiani, Nghiep Khoan Duong, Quynh P. Sam, Han Wang, Yeryun Cheon, David J. Hynek, Hyeuk Jin Han, Ravishankar Sundararaman, Judy J. Cha

**Affiliations:** 1 Department of Electronic Engineering, Gachon University, Seongnam, 13120 South Korea; 2 Department of Materials Science and Engineering, Rensselaer Polytechnic Institute, Troy, NY 12180 USA; 3 Department of Materials Science and Engineering, Cornell University, Ithaca, NY 14850 USA; 4 Department of Physics, Cornell University, Ithaca, NY 14850 USA; 5 Department of Mechanical Engineering and Materials Science, Yale University, New Haven, CT 06511 USA; 6 Department of Environment and Energy Engineering, Sungshin Women’s University, Seoul, 01133 South Korea

**Keywords:** Synthesis and processing, Electronic properties and materials

## Abstract

Topological materials confined in 1D can transform computing technologies, such as 1D topological semimetals for nanoscale interconnects and 1D topological superconductors for fault-tolerant quantum computing. As such, understanding crystallization of 1D-confined topological materials is critical. Here, we demonstrate 1D template-assisted nanowire synthesis where we observe diameter-dependent phase selectivity for tungsten phosphides. A phase bifurcation occurs to produce tungsten monophosphide and tungsten diphosphide at the cross-over nanowire diameter regime of 35–70 nm. Four-dimensional scanning transmission electron microscopy is used to identify the two phases and to map crystallographic orientations of grains at a few nm resolution. The 1D-confined phase selectivity is attributed to the minimization of the total surface energy, which depends on the nanowire diameter and chemical potentials of precursors. Theoretical calculations are carried out to construct the diameter-dependent phase diagram, which agrees with experimental observations. Our findings suggest a crystallization route to stabilize topological materials confined in 1D.

## Abstract (web summary)

Topological materials confined in 1D could transform computing technology, but their crystallization is poorly understood. Here, the authors demonstrate template-based synthesis of 1D nanowires, revealing diameter-dependent phase selectivity.

## Introduction

Nanostructured transition-metal phosphides (TMPs) are a promising material platform for energy storage, catalysis, and photonics^1–6^. A subset of TMPs possess topologically non-trivial electronic band structures: group V phosphides such as niobium phosphide and tantalum phosphide are Weyl semimetals^7–9^, and group VI phosphides such as molybdenum monophosphide (MoP) and tungsten monophosphide (WP) are topological metals that exhibit high conductivity and high carrier density, along with the topologically protected fermions^10–13^. Additional interesting properties for WP include superconductivity with a small electron-phonon coupling strength^14–17^ and multiple semi-Dirac-like points near the Fermi level^17^. Tungsten diphosphide (WP_2_) is another topological TMP that exhibits high magnetoresistance (3 × 10^5^%) due to compensated semimetal characteristics and large suppression of backscattering which can be attributed to the robust topological phase with two neighboring Weyl points^18–20^. These TMP topological semimetals^8–20^ are an emerging class of promising nanoscale interconnect materials, especially when confined in 1D, which can potentially deliver the desired dimensional scaling of decreasing resistivity with decreasing dimensions, arising from the topological surface states and suppressed electron backscattering^21–24^. An anisotropic conductor within a 1D framework is also a promising interconnect metal if the high fermi velocity direction is oriented along the length of the 1D wire such that surface scattering is greatly suppressed^25^. In the context of classical expression, the resistivity of metal wires increases as their width decreases below the bulk electron mean free path (*λ*). This increase in resistivity is due to contributions from surface and grain-boundary scattering^25–28^.

Despite the promises for low-resistance nanoscaled interconnects, controlled synthesis of nanostructured topological semimetals has been under-investigated. Nevertheless, precision synthesis of 1D-confined TMPs is essential for the realization of energy-efficient computing technologies based on their emergent transport phenomena.

For crystallization in 1D, the free-energy landscape, which underlies the kinetics and thermodynamics of the crystallization process, is significantly affected by the nanoscale confinement^29–31^. At large surface-to-volume ratios, surface energy of the growth products can dominate the crystallization pathway: a metastable phase might be preferred over a stable phase if the surface energy of the metastable phase is lower. Thus, nanoscale confinement can be exploited to control phase stability and synthesis pathways^32,33^. A 2D-confined template is commonly used to achieve transition-metal nitrides and transition-metal phosphides in recent reports. This method ensures a homogeneous phase across the unconventional 2D structures, regardless of the template’s thickness, e.g., MoS_2_, TiS_2_, or WS_2_^13,34^. For these crystallization in confined dimensions, atomistic understanding can be further developed to achieve phase selectivity via geometric confinement.

Here, we developed a 1D-confined synthesis of topological metal WP and WP_2_ via 1D template-assisted transformations. With decreasing diameter, we observe crystallization pathways, resulting in distinct phases. The size effects on phase stability of tungsten phosphides were studied using four-dimensional scanning transmission electron microscopy (4D-STEM) and automated crystal orientation mapping (ACOM) on 4D STEM datasets^35,36^. Diameter-dependent phase selectivity of WP or WP_2_ is attributed to the surface-energy differences of the synthesized nanowires. A diameter-dependent phase diagram was constructed by density-functional-theory (DFT) calculations, which support the observed WP and α-WP_2_. For the interconnect applications, we measured resistivity of the synthesized nanowires and show that polycrystalline 1D-WP should have minimized surface electron scattering due to its small mean free path of 3.15 nm. Our findings demonstrate that diameter-dependent crystallization is a viable synthesis route for 1D topological semimetals.

## Results

### Band structure and synthesis of 1D tungsten phosphide

**Fig. 1:**
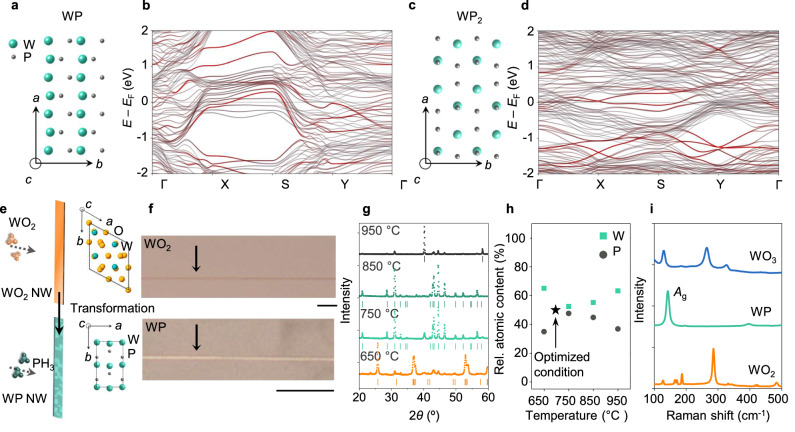
Electron band structures and template-assisted conversion of WP and WP_2_. Crystal structure (**a**) and calculated surface-weighted electronic band structure (**b**) of a WP slab, terminated with the lowest surface energy plane (011). Surface states from the terminated crystal planes are colored in red. Note that these surface states do not represent topologically protected surface states. Crystal structure (**c**) and calculated surface-weighted electronic band structure (**d**) of a α-WP_2_ slab, terminated with the lowest surface energy plane (110). Surface states from the terminated crystal planes are colored in red. We have chosen the same high-symmetry path for orthorhombic WP and monoclinic WP_2_ since these band structures represent slabs rather than bulk structures, and the slabs have identical surface unit cells. **e** Schematics of geometrically confined transformation from WO_2_ to WP with corresponding crystal structures of WO_2_ and WP. **f** Optical microscope images of WO_2_ template grown on c-cut sapphire and transformed 1D-WP. scale bars: 10 μm. **g** Powder X-ray diffraction spectra of growth products as a function of the conversion temperature (orange, 650 °C; light green, 750 °C; dark green, 850 °C; black, 950 °C). **h** Relative atomic content of W_*x*_P_1-*x*_ (0.5 < *x* < 0.7) growth products, collected from SEM-EDS signals at 4 different conversion temperatures. **i** Raman spectra of as grown 1D-WO_2_ (orange), transformed 1D-WP (light green), and WO_3_ (blue), respectively.

DFT calculations were performed to obtain electronic band structures of WP and WP_2_. WP has an orthorombic crystal structure (Fig. 1a) with lattice parameters *a* = 0.327 nm, *b* = 0.576 nm, *c* = 0.627 nm^17^. To represent nanoscale WP with large surface-to-volume ratios, we calculated a surface-weighted band structure of an 8-unit cell thick WP slab that was terminated with the lowest surface energy crystal planes of (011) (Fig. 1b). Monoclinic α-WP_2_ with lattice parameters *a* = 0.848 nm, *b* = 0.319 nm, *c* = 0.748 nm is one of the stable W-P compounds at room temperature (Fig. 1c). α-WP_2_ is topologically trivial unlike β-WP_2_ that has Weyl nodes^37^. The surface-weighted band structure of a monoclinic 11-unit cell thick α-WP_2_ slab terminated with (110) crystal planes is shown in Fig. 1d. Band structure calculations show both are metals.

Highly anisotropic WP nanostructures were synthesized via 1D-confined transformation by converting WO_2_ nanowires to WP nanowires, as illustrated in Fig. 1e. First, we grew 1D-WO_2_ on the *c*-sapphire substrates with a miscut angle of 1° along the *a*-axis of sapphire (〈11–20〉)^38,39^ by vapor transport synthesis from WO_3_ powder precursors. As-grown 1D-WO_2_ templates (monoclinic, *P*2_1_/*c* space group, *a* = 0.576 nm, *b* = 0.484 nm, *c* = 0.580 nm) were transformed to orthorhombic WP via phosphorization using PH_3_ gas, produced from the thermal decomposition of NaH_2_PO_2_·H_2_O at 700 °C (see Methods and Supplementary Fig. 1 for growth aspects). The transformed WP exhibits a highly directional crystalline form with typical widths of 100–500 nm and lengths of 10–300 μm (aspect ratio of ≈ 100) as shown in Fig. 1f and Supplementary Fig. 2. The conversion from WO_2_ to WP is accompanied by the volume decrease from the unit-cell volume of 149.31 Å^3^ for WO_2_ to 117.90 Å^3^ for WP due to the change in crystal symmetry and lattice parameters. The WO_2_ to WP transformation is apparent by the color change in converted crystals, but the original morphologies of the WO_2_ nanowires were preserved in converted WP.

The conversion temperature was optimized by analyzing X-ray diffraction (XRD) and energy-dispersive X-ray spectroscopy (EDS) data (Fig. 1g, h). At 650 °C, the powder product still contained WO_2_ diffraction peaks and excess W with an elemental ratio of W:P = 1.86:1, which suggest incomplete conversion to WP. At higher temperatures (>750 °C), XRD patterns mainly indicate the orthorhombic WP without the formation of other W-P phases, such as α-WP_2_, β-WP_2_, WP_3_, or W_3_P. However, the stoichiometry of converted WP deviated from the expected W:P ratio of 1:1. With increasing conversion temperature, the W:P ratio was 1.1:1 at 750 °C, 1.2:1 at 850 °C, and 1.7:1 at 950 °C. From the XRD and EDS analysis, the conversion temperature was set to 700 °C to achieve the W:P ratio of 1:1 (magnified XRD spectra in Supplementary Fig. 3). SEM-EDX analysis of individual wires are shown in Supplementary Fig. 4. To verify the formation of WP and absence of any residual tungsten oxides, we also obtained the Raman spectra (excitation 532 nm) of initial WO_2_ templates and final WP nanostructures converted at 700 °C (Fig. 1i and Raman map in Supplementary Fig. 5). The Raman spectrum of the WO_2_ template (orange) is in agreement with previous studies of 1D WO_2_^40^. The Raman spectrum of the WP nanostructures (light green) shows a strong scattering peak at 142.3 cm^−1^, indicating the *A*_g_ vibration mode^15^. At a higher laser intensity (>300 μW), the *A*_g_ peak disappeared and the WP nanostructures were oxidized to WO_3_, as supported by the presence of the Raman peaks (blue) at 130, 264, and 326 cm^−1^. Thus, we confirm the complete transformation to WP from WO_2_ at the conversion temperature of 700 °C based on XRD, EDS, and Raman spectroscopy.

### Structure characterization of 1D-WP with varying diameter

**Fig. 2:**
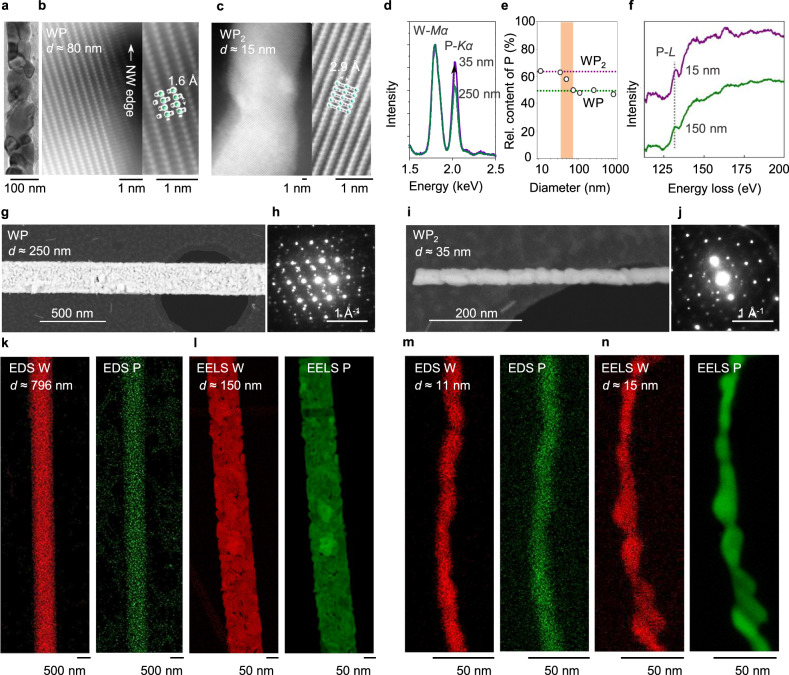
Structural characterization of 1D-confined WP_*x* (*x* = 1 or 2)_ with varying diameter. **a** Low-magnification TEM image of 1D-confined WP. Atomic-resolution HAADF-STEM and magnified images of WP (**b**) and WP_2_ (**c**) obtained from a single grain of the polycrystalline WP and WP_2_ nanowires. **d** STEM-EDS spectra acquired from representative 1D-confined WP_*x* (*x* = 1 or 2)_ (green, 250 nm in diameter; purple, 35 nm in diameter). **e** Relative atomic content of P with varying diameter, collected from TEM-EDS signals of 7 different samples. Cross-over regime is 35–70 nm. **f** EELS P *L*-edge of 1D-confined WP_*x* (*x* = 1 or 2)_ (green, 150 nm in diameter; purple, 15 nm in diameter). HAADF-STEM images of 1D-confined WP (**g**) with the diffraction pattern of dominant zone axis of [131] (**h**) and WP_2_ (**i**) with the corresponding diffraction pattern of zone axis of [233] (**j**). False-color STEM-EDS maps (W-*L* in red, P-*K* in green) (**k**) and EELS maps (W-*O* in red, P-*L* in green) (**l**) obtained from two WP nanowires. False-color STEM-EDS maps (W-*L* in red, P-*K* in green) (**m**) and EELS maps (W-*O* in red, P-*L* in green) (**n**) collected from two WP_2_ nanowires. The W:P ratio of the WP nanowire (diameter ≈ 796 nm) shown in (**k**) is 0.53:0.47 and the ratio of the WP_2_ nanowire (diameter ≈ 11 nm) shown in (**m**) is 0.36:0.64.

Using transmission electron microscopy (TEM), we characterized the atomic structure of the 1D-confined WP with various diameters. The TEM image presented in Fig. 2a shows the polycrystalline nature of the 1D-confined WP with nanoscale grains. Individual WP grains are merged to form a closed-packed nanowire (width of ≈ 100 nm) without any pores and noticeable oxide layers. The high-angle annular dark-field scanning transmission electron microscopy (HAADF-STEM) image of one of the grains in the nanowire confirms the atomic structure of WP with bright W atomic columns that arise from the atomic number (*Z*) difference of *Z*_P_ = 15 and *Z*_W_ = 74. The lattice image of the transformed WP agrees with the atomic model viewed along the [001] direction of WP (green: W atoms, grey: P atoms) (Fig. 2b). This is distinguishable from the atomic-resolution HAADF-STEM image of WP_2_ and corresponding atomic model (Fig. 2c).

The chemical compositions of the nanowires were analyzed by STEM-EDS (Fig. 2d, e), which showed that a 35 nm-wide nanowire had significantly less P by 63–64% compared to a 250 nm-wide nanowire, which showed roughly 1:1 atomic ratio of W:P. Several nanowires were analyzed using STEM-EDS and showed that below ≈50 nm diameter, the W:P ratio started to deviate from 1:1. This suggests the growth products might contain more than one phase of W-P compounds despite our bulk XRD analysis that only showed the monoclinic WP phase (Fig. 1g). The electron energy loss spectroscopy (EELS) spectra taken from 15-nm-diameter nanowire and 150-nm-diameter nanowire show the P *L*-edge (Fig. 2f) (for more detailed EELS data, refer to Supplementary Fig. 6). Micro-structures of two samples with different diameters were examined using HAADF-STEM (≈250 nm diameter in Fig. 2g, h and 15–35 nm diameter in Fig. 2h, j and Supplementary Fig. 7), which showed increasing porosity and more grains with increasing diameter. Surprisingly, the electron diffraction patterns from the 35-nm- and 250-nm-wide nanowires were different, which could not simply be attributed to different tilt angles of the nanowires with respect to the electron beam. STEM-EDS maps and EELS maps for 1D-WP (Fig. 2k, l) and 1D-WP_2_ (Fig. 2m, n) clearly show the differences in the size of nanowires.

### Crystallographic orientation mapping of WP and α-WP_2_

**Fig. 3:**
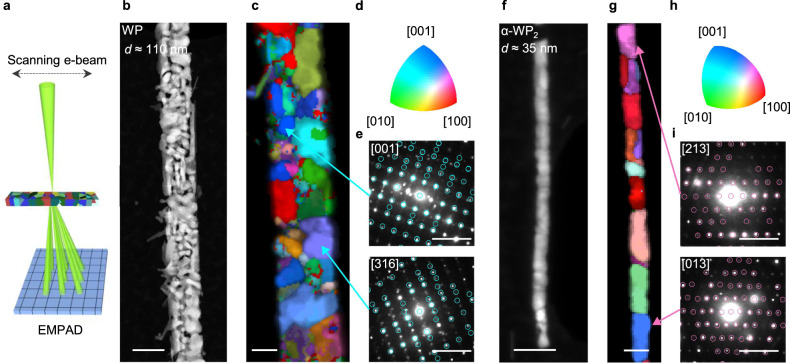
4D STEM and grain orientation mapping of WP and α-WP_2_. **a** Schematic for 4D-STEM of 1D-confined WP_*x* (*x* = 1 or 2)_ on EMPAD. HAADF-STEM image (**b**) and grain orientation map (**c**) of 1D-confined orthorhombic WP. The orientation map only shows a sub-section of the wire. Each color represents a crystal plane corresponding to the color-coded inverse pole figure shown in (**d**). Inverse pole figure for WP (**d**) and electron diffraction patterns (**e**) with simulated diffraction patterns from specific grains of orthorhombic WP. HAADF-STEM image (**f**) and grain orientation map (**g**) of 1D-confined monoclinic WP_2_. Each color in a grain orientation map represents a crystal plane corresponding to the color-coded inverse pole figure shown in (**h**). Inverse pole figure for WP_2_ (**h**) and electron diffraction patterns (**i**) with simulated diffraction patterns from specific grains of monoclinic WP_2_. Scale bar is 1 Å^−1^. Scale bars: 100 nm for (**b**) and (**f**), 50 nm for (**c**) and (**g**), 1 Å^−1^ for (**e**) and (**i**). For the overlapping experimental and simulated diffraction patterns, the label provides the nearest zone axis.

To discern additional phases beside WP in the converted phosphides, we carried out 4D-STEM on converted phosphide nanowires to obtain crystal structures of individual grains as illustrated in Fig. 3a (see Methods). With the 4D-STEM method, the electron beam is focused to a nanoscale probe, rastered across the sample surface, and a full diffraction pattern is collected at each spatial coordinate. These diffraction patterns can then be processed to determine the local phase. To do so, we use the ACOM package with py4DSTEM^35,36^. With this analysis method (Supplementary Fig. 8), we compare the experimental diffraction data with simulated diffraction patterns of WP, α-WP_2_, and β-WP_2_ for all possible orientations, which allows the determination of the crystal phase as well as the crystal orientation. Figure 3b shows a 110-nm-wide tungsten phosphide nanowire; our 4D-STEM analysis indicates that this nanowire is predominantly orthorhombic WP (space group of *Pnma*). The WP crystal orientation map shows that the nanowire is polycrystalline, with random grain orientations (Fig. 3c, d). Note that for this specimen, the domain size is smaller than the nanowire thickness (in the beam direction), such that all the recorded diffraction patterns contain signals from multiple grains. Accordingly, the orientation maps reflect the orientation of the grain which produces the highest diffraction intensity, and the highest correlation with the simulated patterns (Fig. 3e). In contrast, a 35-nm-wide tungsten phosphide nanowire shown in Fig. 3f was identified to be predominantly monoclinic α-WP_2_ (space group of *C*2/*m*) according to the 4D STEM data and subsequent analysis. This can explain the deviation in the W:P ratio of the nanowires with diameter below ≈50 nm analyzed by TEM-EDS (Fig. 2d), which showed a W:P ratio of ~1:2. The grain orientation map of the α-WP_2_ nanowire (Fig. 3g, h) shows random grain orientations (Fig. 3i). Thus, using 4D STEM, we observed diameter-dependent phase bifurcation in the crystallization of 1D-confined tungsten phosphides where WO_2_ is converted to WP or WP_2_ above and below the diameter range of 35–70 nm, respectively. These two phases for the different diameter nanowires were also confirmed with TEM-EDS (full list of material characterizations and their sample scales for α-WP_2_ and WP is provided in Supplementary Fig. 9).

### Size-dependent phosphorization pathways of WP and α-WP_2_

**Fig. 4:**
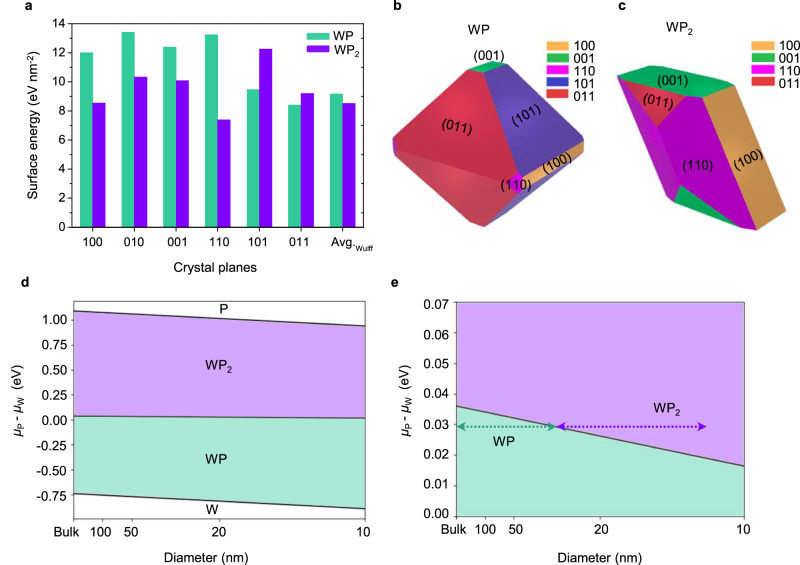
Size-dependent crystallization pathways of WP and α-WP_2_. **a** Calculated surface energies of various crystal planes for WP (green) and α-WP_2_ (purple). Wulff polyhedron constructions for WP (**b**) and WP_2_ (**c**) (yellow, 100 plane; green, 001 plane; magenta, 110 plane; blue, 101 plane; red, 011 plane). **d** Phase diagram for tungsten and phosphorus as a function of diameter from bulk to 10 nm. **e** Enlarged phase diagram for tungsten and phosphorus as a function of diameter. Arrows are estimated experimental conditions showing the transition of the phase at ~35 nm.

Theoretical calculations were carried out to understand the experimentally observed diameter-dependent phases. We first calculated surface energies of various crystal planes for the two distinct phases of WP and α-WP_2_ (Fig. 4a and Supplementary Table 1) and then constructed appropriate Wulff shapes to minimize the total surface energy for each phase (Fig. 4b, c). Since we observed polycrystalline textures for each phase from the grain orientation maps (Fig. 3), the most stable crystallographic planes were averaged. We found that the average surface energy of the α-WP_2_ Wulff shape (8.516 eV nm^−2^) is lower than that of the WP Wulff shape (9.162 eV nm^−2^), which suggests that α-WP_2_ should be preferred over WP with decreasing diameter that corresponds to increasing surface-to-volume ratio. The surface energy comparison is consistent with our experimental observation that α-WP_2_ is observed in nanowires with diameter below ≈35 nm and WP above ≈35 nm.

To quantify this phase transition, we compute the free energy, *g*_WP_, of WP and WP_2_ phases. In doing so, surface energy becomes a crucial parameter when assuming cylindrical nanowire geometry. We normalize this free energy per atom to allow for a direct comparison between materials, which provides the equation:

1 $${g}_{{{{\rm{WP}}}}}={E}_{{{{\rm{Formation}}}},{{{\rm{WP}}}}}-\frac{1}{2}{\mu }_{{{{\rm{PW}}}}}+\frac{4{E}_{{{{\rm{Surface}}}}}{v}_{{{{\rm{WP}}}}}}{d}$$

where *E*_Surface_ is the average surface energy per unit area as calculated from the Wulff model, *v*_WP_ is the unit cell volume per atom, *d* is the nanowire diameter, and *μ*_PW_ = *μ*_P_–*μ*_W_ is the chemical potential difference between P and W. In Eq. (1), the factor of 1/2 corresponds to the mole fraction of P in WP. To obtain the corresponding equation for WP_2_, this fraction would simply be modified to 2/3. For both phases, formation energy was taken from the Materials Project database^41^. A phase diagram was calculated as a function of *μ*_PW_ and *d* by equating *g*_WP_ and *g*_WP2_ (Fig. 4d, e), which predicts a transition of stable phase from WP to α-WP_2_ with decreasing diameter. We note that we had previously studied topological metal MoP nanowires using the same template-assisted growth method^11^ and accordingly construct a diameter-dependent phase diagram for MoP (Supplementary Fig. 10), which shows a different behavior from the W-P phase diagram.

### Electron-transport properties of 1D-confined WP

**Fig. 5:**
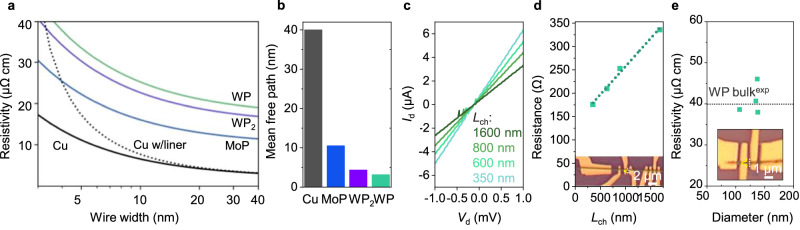
Resistivity scaling of WP. **a** Calculated resistivity scaling of topological metal phosphides (WP (green), WP_2_ (purple), MoP (blue)) as square wires, in comparison with Cu square wires with (dotted black) and without liner (black). The liner was assumed to be 2 nm thick. Single crystal was assumed for the calculations with complete diffuse electron scattering at surfaces (*p* = 0). We note that experimental bulk resistivities (*r*_0_) are used when width → ∞. **b** Calculated average mean free paths of Cu (black), MoP (blue), WP_2_ (purple), and WP (green). **c** Channel-length (*L*_ch_) dependent current-voltage (I-V) curves of the 1D-confined polycrystalline WP (turquoise, 350 nm; light green, 600 nm; green, 800 nm; dark green, 1600 nm). **d** *L*_ch_ dependent resistance variation of the 1D-confined WP. Inset: optical microscopy image and AFM line profile of the measured device. **e** Room-temperature resistivity data of 1D-confined WP with varying cross-sectional area. Dotted line: resistivity value of WP bulk single crystal^16^.

As discussed in the introduction, a subset of TMPs are topological metals that are promising as nanoscaled interconnects. To test the feasibility of these TMPs as interconnects beyond the current 3-nm technology nodes with the metal pitch of ≈24 nm^42^, room temperature resistance of these TMPs at the nanoscale must be obtained. Size-dependent room-temperature resistivities of MoP, WP, and WP_2_ were calculated for two representative geometries of square cross-section wires (Fig. 5a) and thin films (Supplementary Fig. 11) using Fuchs–Sondhemier models to assess electron scattering at surfaces (calculation details in Supplementary Table 2)^43,44^. To minimize the surface scattering, the transport directions were chosen to be along the *a*- and *c*-axes for WP and WP_2_, and the c-axis for MoP given their anisotropic Fermi surfaces (Supplementary Table 3). For comparison, resistivities of Cu wires with and without a liner are also shown in Fig. 5a. For each resistivity curve, complete diffuse surface scattering was assumed with the specularity parameter *p* = 0^45–47^. We did not consider grain-boundary scattering in these calculations because the calculated mean free paths of WP (3.15 nm) and WP_2_ (4.33 nm) (Fig. 5b) were much smaller than the observed grain sizes (Fig. 3). The dimensional scaling of resistivity for WP and α-WP_2_ appears similar to that of Cu without the liner and better than Cu with the liner. However, since the bulk resistivity of WP and WP_2_ is higher than Cu, WP and WP_2_ do not appear promising as low-resistance interconnects. Further, the resistivity scaling calculations suggest that for WP and WP_2_, suppressed electron scattering originating from topologically protected surface states is negligible at room temperature, unlike the cases for CoSi and NbAs^23,24^.

We carried out room-temperature resistivity measurements on the WP nanowires to experimentally gauge the degree of surface and grain boundary scattering as compared to the calculations. Using the transfer length methods, linear *I*–*V* curves were obtained by varying the channel length (Fig. 5c), where the channel resistance increased linearly with the channel length as expected and the contact resistance of 68.9 Ω was extracted (Fig. 5d). Four-probe resistance measurements were carried out on several WP nanowires (Fig. 5e) (two-probe measurement on WP_2_ nanowire is shown in Supplementary Fig. 12). The resistivity values of our 1D-confined polycrystalline WP, which ranged between 38 and 46 μΩ cm for cross-section areas of 8000−15,000 nm^2^ (Supplementary Fig. 13), are comparable to that of bulk WP single crystal (40 μΩ cm) grown by chemical vapor transport^16^. Supplementary Fig. 14 shows the correlation between the resistivity and grain structures for WP nanostructures with TEM images. Thus, in agreement with the calculations, the resistivity of 1D-WP implies that electron scattering at grain boundaries is negligible, which can be attributed to the small mean free path (3.15 nm).

## Discussion

We have demonstrated size-dependent phosphorization routes of 1D-confined WP and WP_2_ from 1D WO_2_ templates. Crystallographic orientations of grains and grain distributions for each phase were characterized using 4D-STEM, revealing critical transition regime of 35–70 nm diameter below and above which WP_2_ and WP form. The calculated surface energies from the Wulff constructions of these two competing phases predict α-WP_2_ to be stable over WP at the nanoscale, which was further shown in the diameter-dependent phase diagrams obtained by DFT calculations. We achieved the lowest resistivity of 38 μΩ cm for WP nanostructures, which is comparable to that of bulk single crystal, indicating electron scattering at grain boundaries and surfaces must be small despite the disordered polycrystalline nature of our samples likely due to their small mean free path. Our findings suggest that the diameter-dependent crystallization route can be exploited to guide synthesis protocols of 1D topological semimetals.

## Methods

### 1D-confined conversion of WP and WP_2_

1D-WO_2_ were grown by chemical vapor deposition. WO_3_ source powder (Sigma-Aldrich, 99.95%) was placed at the center of the hot-walled tube furnace. Chamber pressure was maintained at 3 torr with 20 cm³ STP min^−1^ of H_2_ gas. C-sapphire substrate with the miscut angle of 1° was placed at upstream of the furnace. The temperature of the upstream was maintained at 600 °C for 10 min. To convert the WO_2_ templates to WP and WP_2_, the as-grown 1D WO_2_ templates were placed in the center of the furnace with 3 g of NaH_2_PO_2_·H_2_O (Sigma-Aldrich, ≥99%) placed upstream. The chamber was pumped down to 100 mtorr, and then 30 cm³ STP min^−1^ of H_2_ was flowed until the furnace pressure reached atmospheric pressure. The temperature of the furnace was ramped up to 700 °C and held there for 50 min.

### 4D-STEM and EELS measurements

STEM experiments were performed on a C_s_-probe-corrected Thermo Fisher Scientific Spectra 300 with an extreme-brightness cold field emission gun. The 4D-STEM measurements were collected using an EMPAD at 120 kV and a convergence angle of 0.5 mrad. The 4D-STEM datasets were processed using py4DSTEM and the associated crystal-orientation mapping code^35,36^ EELS measurements were conducted using an aberration-corrected TFS Titan Themis 300 X-FEG, equipped with a Gatan GIF Tridiem energy filter. The microscope was operated at 120 kV, with *a* < 100 pA beam current and a convergence semi-angle of 5 mrad. The stoichiometric ratio of W:P was determined from EDX analysis. From EELS, it is difficult to obtain the composition because of the background subtraction issue. For W, we obtained the W-O edge, which is at 47 eV, since the W-M edge at 1800 eV yields signals that are too weak. The W-O edge is too close to the bulk plasmon, making the power-law-type background subtraction inaccurate. Thus, we cannot obtain the stoichiometric ratio from EELS.

### DFT calculations

All first-principles calculations were performed using the open-source plane-wave software JDFTx^48^. Calculations for all materials were run using the Perdew–Burke–Ernzerhof (PBE) exchange-correlation functional^49^ with a plane wave energy cutoff of 680 eV, and ultrasoft pseudopotentials were sourced from the GBRV library^50^. Self-consistent calculations were performed using a Gamma-centered mesh of 12 × 12 × 12 *k*-points to compute the bulk free energies of WP and WP_2_, whereas surface-oriented slabs utilized a 12 × 12 × 1 *k*-point mesh. Slab geometries were constructed such that the slab thickness was ~30 Å, with a vacuum spacing of 15 Å. A structural relaxation was performed iteratively for bulk and slab structures to optimize the lattice constants and atomic positions. Upon calculating surface energies for WP and WP_2_, Wulff models were constructed using the WulffPack Python package^51^. For the calculation of bulk resistivities, all electronic structure and phonon properties were transformed into the maximally localized Wannier function basis^52^. In the case of WP, a total of 48 Gaussian Wannier centers were iteratively fitted to the band structure in the energy range −15.3 eV to +4.55 eV, relative to the VBM, and a phonon *q*-mesh of 4 × 2 × 2 was chosen. For WP_2_, a total of 48 Gaussian Wannier centers were fitted in the energy range −16.9 eV to +4.38 eV, and a phonon *q*-mesh of 2 × 4 × 2 was chosen.

### Device fabrication of 1D-confined WP

The converted WP crystals were transferred onto SiO_2_/Si substrates using a PMMA-assisted wet-transfer method, then coated with e-beam resist layers (200 nm MMA EL 8.5 and 200 nm PMMA A3). Electrode patterns for transfer-length methods and four-probe measurements were written by standard e-beam lithography using a ThermoFisher Helios G4 system. 10/100 nm-thick Cr/Au electrical contacts were deposited by UHV e-beam evaporation followed by in-situ Ar etching (50 W).

### Reporting summary

Further information on research design is available in the Nature Portfolio Reporting Summary linked to this article.

### Supplementary information

Supplementary Information

Peer Review File

Reporting Summary

## Data Availability

The data that support the findings of this study are available from the corresponding authors upon request.

## References

[1] Li S-H, Qi M-Y, Tang Z-R, Xu Y-J (2021). Nanostructured metal phosphides: from controllable synthesis to sustainable catalysis. Chem. Soc. Rev..

[2] Downes CA (2022). Controlled synthesis of transition metal phosphide nanoparticles to establish composition-dependent trends in electrocatalytic activity. Chem. Mater..

[3] Tang Z (2020). Phosphorus science-oriented design and synthesis of multifunctional nanomaterials for biomedical applications. Matter.

[4] Zong Q, Liu C, Yang H, Zhang Q, Cao G (2021). Tailoring nanostructured transition metal phosphides for high-performance hybrid supercapacitors. Nano Today.

[5] Won Y-H (2019). Highly efficient and stable InP/ZnSe/ZnS quantum dot light-emitting diodes. Nature.

[6] Cossairt BM (2016). Shining light on indium phosphide quantum dots: understanding the interplay among precursor conversion, nucleation, and growth. Chem. Mater..

[7] Kumar N, Guin SN, Manna K, Shekhar C, Felser C (2021). Topological quantum materials from the viewpoint of chemistry. Chem. Rev..

[8] Xu S-Y (2015). Experimental discovery of a topological weyl semimetal state in TaP. Sci. Adv..

[9] Shekhar C (2015). Extremely large magnetoresistance and ultrahigh mobility in the topological weyl semimetal candidate NbP. Nat. Phys..

[10] Lv BQ (2017). Observation of three-component fermions in the topological semimetal molybdenum phosphide. Nature.

[11] Han HJ (2023). Topological metal MoP nanowire for interconnect. Adv. Mater..

[12] Kumar N (2019). Extremely high conductivity observed in the triple point topological metal MoP. Nat. Commun..

[13] Wang W (2022). Preparation of 2D molybdenum phosphide via surface-confined atomic substitution. Adv. Mater..

[14] Cuono G (2019). Multiple band crossings and fermi surface topology: role of double nonsymmorphic symmetries in MnP-type crystal structures. Phys. Rev. Mater..

[15] Zhang Y (2022). Single-crystalline transition metal phosphide superconductor WP studied by Raman spectroscopy and first-principles calculations. Phys. Rev. B.

[16] Liu Z (2019). Superconductivity in WP single crystals. Phys. Rev. B.

[17] Cakmak M, Tayran C (2019). Electronic structure, phonon and superconductivity for WP 5d-transition metal. J. Appl. Phys..

[18] Kumar N (2017). Extremely high magnetoresistance and conductivity in the type-II Weyl semimetals WP_2_ and MoP_2_. Nat. Commun..

[19] Wulferding D (2020). Effect of topology on quasiparticle interactions in the Weyl semimetal WP_2_. Phys. Rev. B..

[20] Gooth J (2018). Thermal and electrical signatures of a hydrodynamic electron fluid in tungsten diphosphide. Nat. Commun..

[21] Lien S-W (2023). Unconventional resistivity scaling in topological semimetal CoSi. NPJ Quantum Mater..

[22] Schmitt AL, Zhu L, Schmeiber D, Himpsel JF, Jin S (2006). Metallic single-crystal CoSi nanowires via chemical vapor deposition of single-source precursor. J. Phys. Chem. B.

[23] Chen, C.-T. et al. Topological semimetals for scaled back-end-of-line interconnect beyond Cu. In Proc. *IEEE International Electron Devices Meeting (IEDM)*, 32.4.1–32.4.4 (IEEE, San Francisco, CA, 2020).

[24] Zhang C (2019). Ultrahigh conductivity in Weyl semimetal NbAs nanobelts. Nat. Mater..

[25] Gall D (2021). Materials for interconnects. MRS Bull..

[26] Wang A-Q, Ye X-G, Yu D-P, Liao Z-M (2020). Topological semimetal nanostructures: from properties to topotronics. ACS Nano.

[27] Liu P, Williams JR, Cha JJ (2019). Topological nanomaterials. Nat. Rev. Mater..

[28] Han HJ, Liu P, Cha JJ (2021). 1D topological systems for next-generation electronics. Matter.

[29] De Yoreo JJ (2015). Crystallization by particle attachment in synthetic, biogenic, and geologic environments. Science.

[30] Yoreo JJD, Vekilov PG (2003). Principles of crystal nucleation and growth. Rev. Mineral. Geochem..

[31] Zhang Z, Lagally MG (1997). Atomistic processes in the early stages of thin-film growth. Science.

[32] Sohn S (2015). Nanoscale size effects in crystallization of metallic glass nanorods. Nat. Commun..

[33] Sohn S, Xie Y, Jung Y, Schroers J, Cha JJ (2017). Tailoring crystallization phases in metallic glass nanorods via nucleus starvation. Nat. Commun..

[34] Cao J (2019). Realization of 2D crystalline metal nitrides via selective atomic substitution. Sci. Adv..

[35] Ophus C (2022). Automated crystal orientation mapping in py4DSTEM using sparse correlation matching. Microsc. Microanal..

[36] Savitzky BH (2021). py4DSTEM: a software package for four-dimensional scanning transmission electron microscopy data analysis. Microsc. Microanal..

[37] Du J (2018). Extremely large magnetoresistance in the topologically trivial semimetal α−WP_2_. Phys. Rev. B.

[38] Jin G (2021). Heteroepitaxial van der Waals semiconductor superlattices. Nat. Nanotechnol..

[39] Jin G (2022). Vapor phase synthesis of topological semimetal MoP_2_ nanowires and their resistivity. Appl. Phys. Lett..

[40] Ma Y-R, Lin C-M, Yeh C-L (2005). Synthesis and characterization of one-dimensional WO_2_ nanorods. J. Vac. Sci. Technol. B.

[41] Jain A (2013). Commentary: the materials project: a materials genome approach to accelerating materials innovation. APL Mater..

[42] Lee Y (2022). Investigation on the effects of interconnect RC in 3nm technology node using path-finding process design kit. IEEE Access.

[43] Fuchs K (1938). The conductivity of thin metallic films according to the electron theory of metals. Math. Proc. Camb. Philos. Soc..

[44] Sondheimer EH (1952). The mean free path of electrons in metals. Adv. Phys..

[45] Josell D, Brongersma SH, Tokei Z (2009). Size-dependent resistivity in nanoscale interconnects. Ann. Rev. Mater. Res..

[46] Adelmann C (2019). On the extraction of resistivity and area of nanoscale interconnect lines by temperature-dependent resistance measurements. Solid-State Electron..

[47] Chawla JS, Gall D (2009). Specular electron scattering at single-crystal Cu(001) surfaces. Appl. Phys. Lett..

[48] Sundararaman R (2017). JDFTx: software for joint density-functional theory. SoftwareX.

[49] Perdew JP, Burke K, Ernzerhof M (1997). Generalized gradient approximation made simple. Phys. Rev. Lett..

[50] Garrity KF, Bennett JW, Rabe KM, Vanderbilt D (2014). Pseudopotentials for high-throughput DFT calculations. Comput. Mater. Sci..

[51] Rahm JM, Erhart P (1944). WulffPack: a Python package for Wulff constructions. J. Open Source Softw..

[52] Marzari N, Vanderbilt D (1997). Maximally localized generalized Wannier functions for composite energy bands. Phys. Rev. B.

