# Supplementary material for: Diameter-dependent phase selectivity in 1D-confined tungsten phosphides
Source: Nat Commun. 2024 Jul 13;15:5889. doi: 10.1038/s41467-024-50323-y (PMC11246448; doi:10.1038/s41467-024-50323-y)
Supplement: Supplementary file 1 — Supplementary Information [file 41467_2024_50323_MOESM1_ESM.pdf]

Supplementary Information

Diameter-dependent phase selectivity in 1D-confined tungsten phosphides

Gangtae Jin,[1] Christian D. Multunas,[2] James L. Hart,[3] Mehrdad T. Kiani,[3] Nghiep Khoan Duong,[4] Quynh P. Sam,[3] Han Wang,[3] Yeryun Cheon,[4] David J. Hynek,[5] Hyeuk Jin Han,[6] Ravishankar Sundararaman [2*], and Judy J. Cha[3*]*

[1] Department of Electronic Engineering, Gachon University, Seongnam, 13120, Republic of Korea

[2] Department of Materials Science and Engineering, Rensselaer Polytechnic Institute, Troy, New York, 12180, USA

[3] Department of Materials Science and Engineering, Cornell University, Ithaca, New York, 14850, USA

[4] Department of Physics, Cornell University, Ithaca, New York, 14850, USA

[5] Department of Mechanical Engineering and Materials Science, Yale University, New Haven, Connecticut 06511, USA

[6] Department of Environment and Energy Engineering, Sungshin Women's University, Seoul, 01133, Republic of Korea

*Corresponding authors:

E-mail: judy.cha@cornell.edu, sundar@rpi.edu, hyeukjin.han@sungshin.ac.kr

Table of Contents

Supplementary Fig. 1. Low magnification optical microscope and SEM images of 1D-confined products. a,b, As-grown 1D-WO_2 templates. **c - h**, Transformed WP and **i,j**, WP_2.

Supplementary Fig. 2. High-aspect ratio 1D-confined WP. a - c, Optical microscope images of representative 1D-transformed WP. **d**, Aspect ratio distribution of WP. **e**, Length as a function of width of WP wires **f**, Thickness as a function of width of WP wires.

Supplementary Fig. 3. Powder X-ray diffraction spectra of growth products as a function of the conversion temperature.

Supplementary Fig. 4. SEM-EDX maps and the corresponding SEM-EDX spectra of 1D-confined WP. a - c , SEM-EDX Maps (**a**) of W (green) (**b**) and P (pink) (**c**). **d,e**, SEM-EDX spectra of P-*K* edge (**d**) and W-*L* edge (**e**).

Supplementary Fig. 5. Raman mapping of 1D-WP and the corresponding Raman spectrum.

Supplementary Fig. 6. EELS spectra of 1D-confined WP and α-WP_2. a, EELS of 150-nm-diameter WP and 15-nm-diameter α-WP_2 showing no noticeable oxidiation regardless of phases. **b**, EELS of the normalized W O-edge of 150-nm-diameter WP and 15-nm-diameter α-WP_2.

Supplementary Fig. 7. HAADF-STEM images from 15-nm-diameter WP_2.

Supplementary Fig. 8. Virtual 4D-STEM annular dark field (ADF) image of a thin nanowire.

Supplementary Fig. 9. List of our material characterizations and their sample scales for α-WP_2 and WP.

Supplementary Fig. 10. A phase diagram was calculated as a function of the chemical potential difference between P and Mo as well as the nanostructure diameter. MoP is preferred over MoP_2 with decreasing diameter.

Supplementary Fig. 11. Size-dependent room temperature resistivities of MoP, WP, and WP_2 calculated for thin film geometries.

Supplementary Fig. 12. Source-drain current (I_{sd}) vs voltage (V_{sd}) for a 35.5 nm α-WP_2 nanowire.

Supplementary Fig. 13. SEM and AFM line profiles for checking diameter and thickness.

Supplementary Fig. 14. Correlation between the resistivity and grain structures for WP nanostructures with corresponding TEM images. a, Room temperature resistivity data of 1D-confined WP with varying cross-sectional area*channel length. **b**, TEM image of 1D-confined WP with varying volumes.

Supplementary Table 1. Calculated surface energy in eV/nm^2 for WP and WP_2.

Supplementary Table 2. Resistivity scaling descriptors: r_{film} and r_{wire}. For definition and computation details, see Suppl. Ref. 1.

Supplementary Table 3. Calculated bulk resistivity values along each crystal axis.

Supplementary References

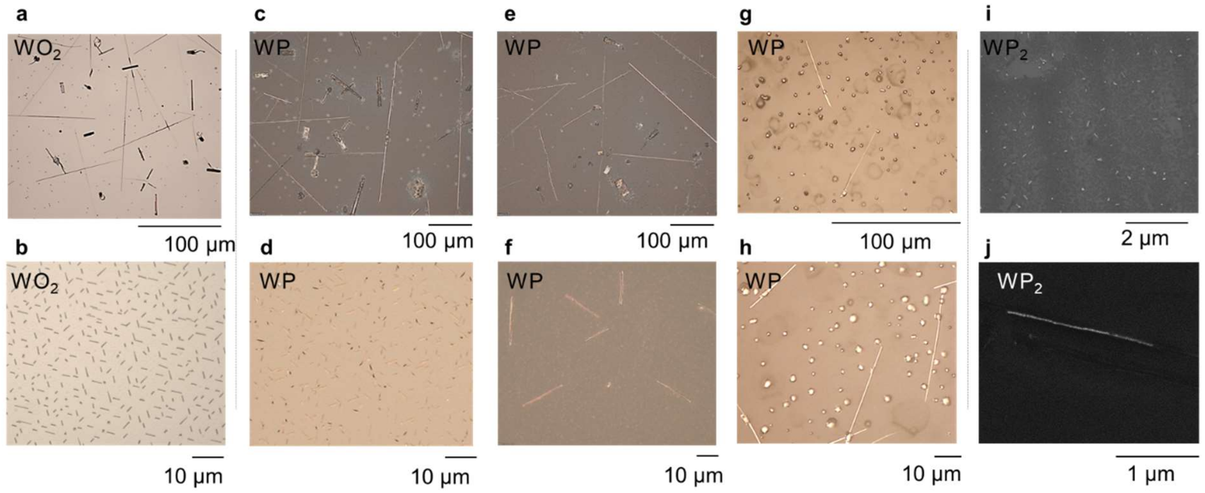

Supplementary Fig. 1. Low magnification optical microscope and SEM images of 1D-confined products. a,b, As-grown 1D-WO_2 templates. **c - h**, Transformed WP and **i,j**, WP_2.

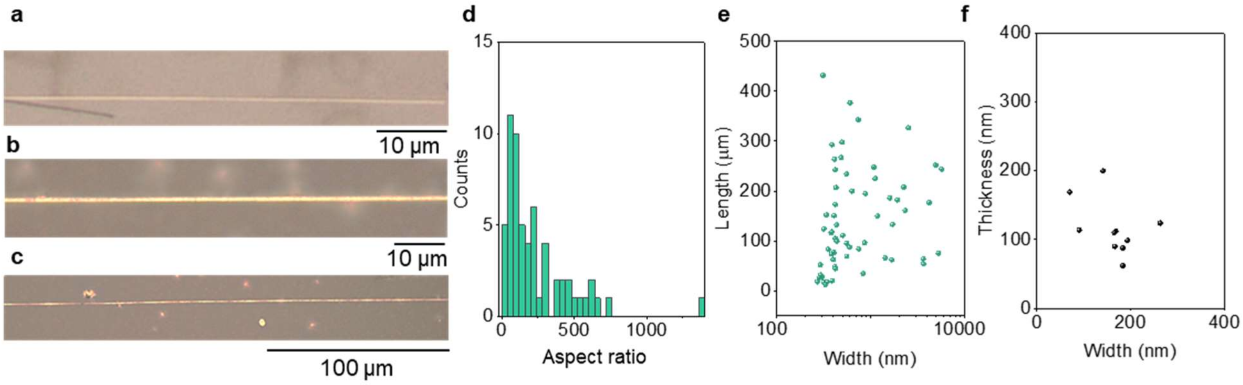

Supplementary Fig. 2. High-aspect ratio 1D-confined WP. a - c, Optical microscope images of representative 1D-transformed WP. **d**, Aspect ratio distribution of WP. **e**, Length as a function of width of WP wires **f**, Thickness as a function of width of WP wires.

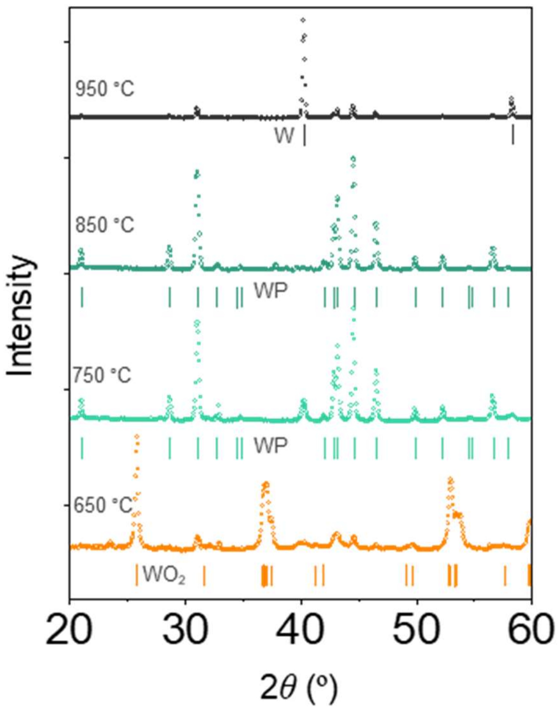

Supplementary Fig. 3. Powder X-ray diffraction spectra of growth products as a function of the conversion temperature.

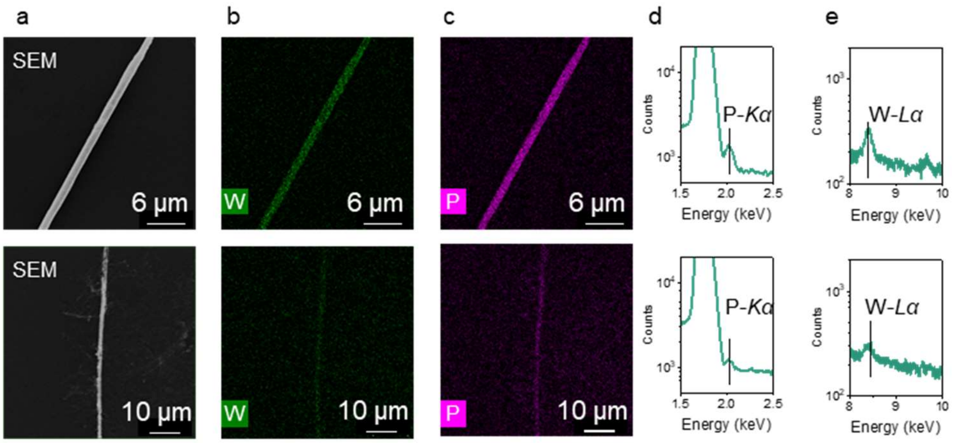

Supplementary Fig. 4. SEM-EDX maps and the corresponding SEM-EDX spectra of 1D-confined WP. a - c , SEM-EDX Maps (**a**) of W (green) (**b**) and P (pink) (**c**). **d,e**, SEM-EDX spectra of P-*K* edge (**d**) and W-*L* edge (**e**).

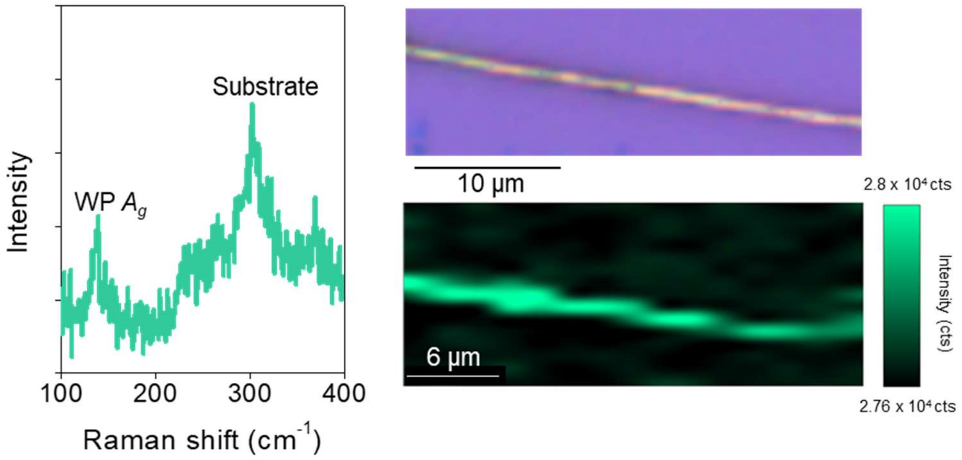

Supplementary Fig. 5. Raman mapping of 1D-WP and the corresponding Raman spectrum.

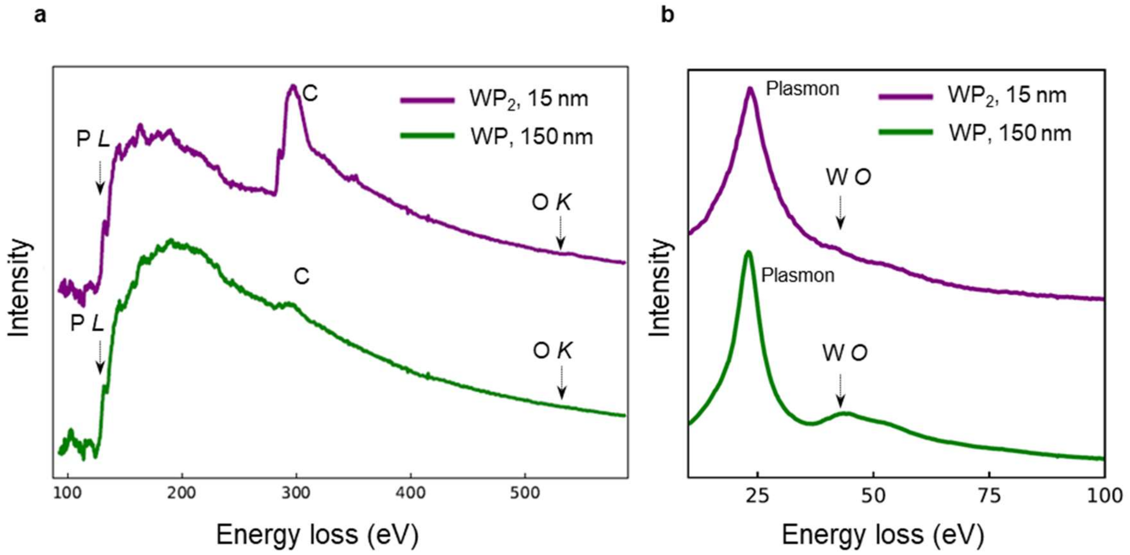

Supplementary Fig. 6. EELS spectra of 1D-confined WP and α-WP_2. a, EELS of 150-nm-diameter WP and 15-nm-diameter α-WP_2 showing no noticeable oxidation regardless of phases. **b**, EELS of the normalized W O-edge of 150-nm-diameter WP and 15-nm-diameter α-WP_2.

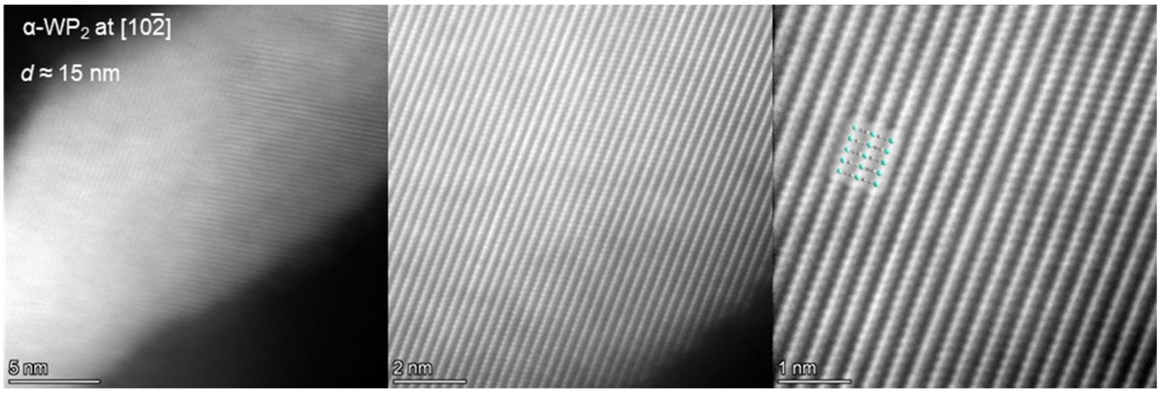

Supplementary Fig. 7. HAADF-STEM images from 15-nm-diameter WP_2.

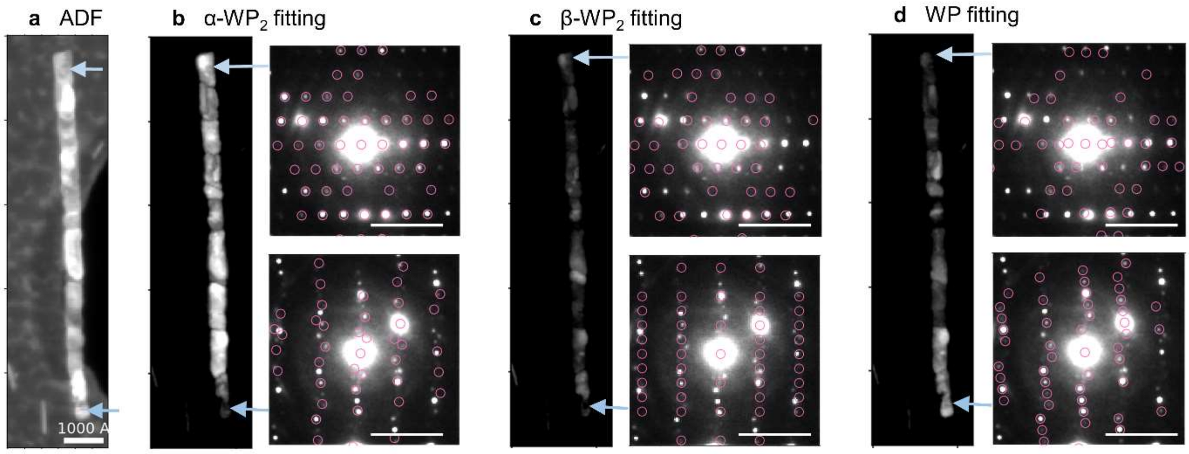

Supplementary Fig. 8. Virtual 4D-STEM annular dark field (ADF) image of a thin nanowire. **a,** virtual 4D-STEM ADF image of the target nanowire. The blue arrows indicate specific grains which we will use as examples for 4D-STEM ACOM processing. **b.** Fitting to the α WP_2 structure. On the left, we show the correlation map for α WP_2. For each pixel in the map, the diffraction pattern is compared against simulations for α WP_2 at all possible orientations. From all of these comparisons, the best fit (with the highest correlation) is selected. The pixel intensity corresponds to the highest correlation value. To the right, we show two example diffraction patterns, with the best fit simulations overlap with the pink circles. Scale bar for diffraction pattern is 1 $Å^{-1}$. **c – d** are the same as **b**, but for β WP_2 and WP, respectively.

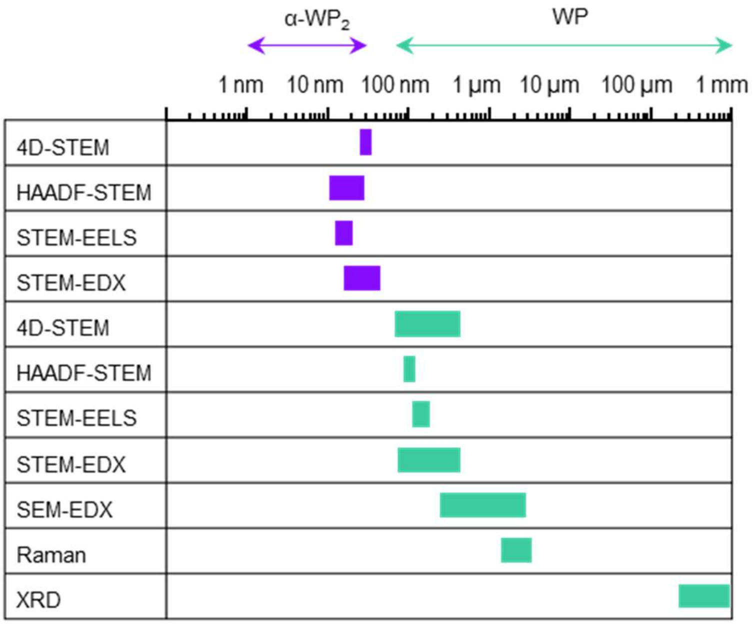

Supplementary Fig. 9. List of our material characterizations and their sample scales for α-WP_2 and WP.

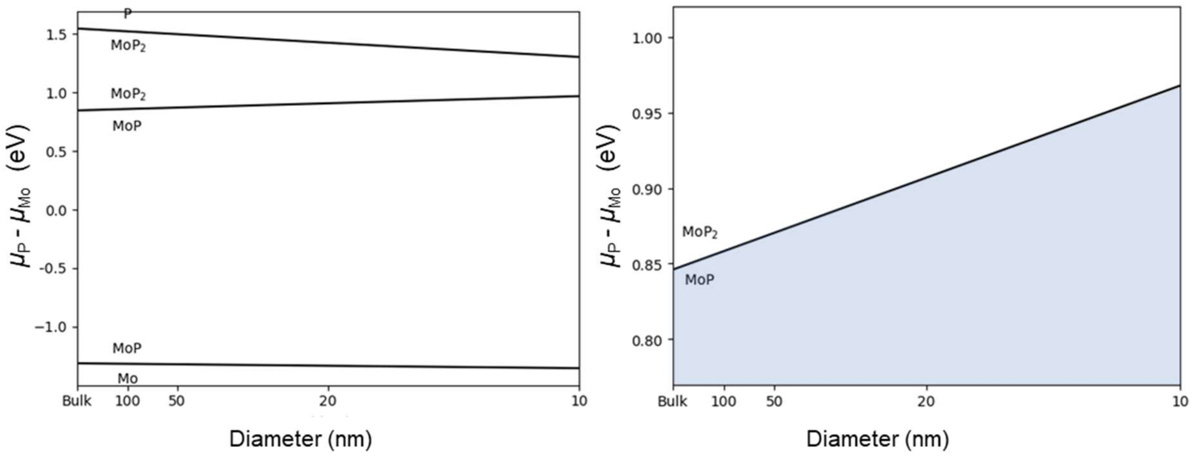

Supplementary Fig. 10. A phase diagram was calculated as a function of the chemical potential difference between P and Mo as well as the nanostructure diameter. MoP is preferred over MoP_2 with decreasing diameter.

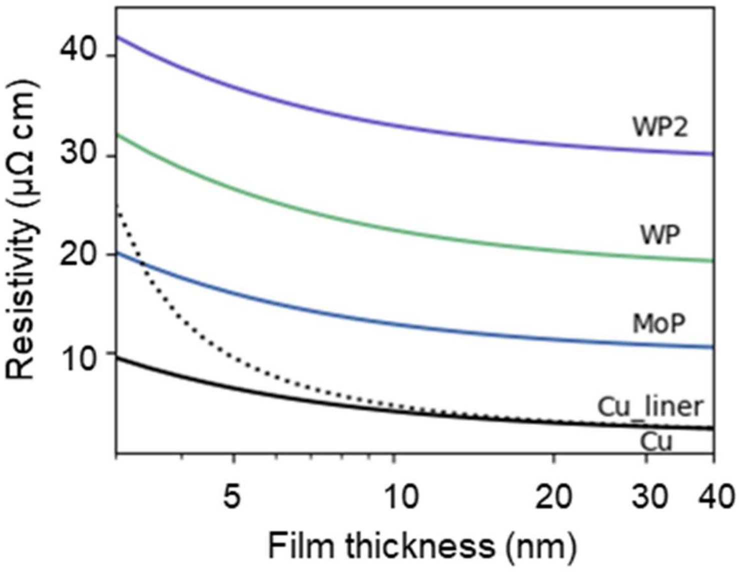

Supplementary Fig. 11. Size-dependent room temperature resistivities of MoP, WP, and WP_2 calculated for thin film geometries. We assumed 2 nm liner for Cu_liner.

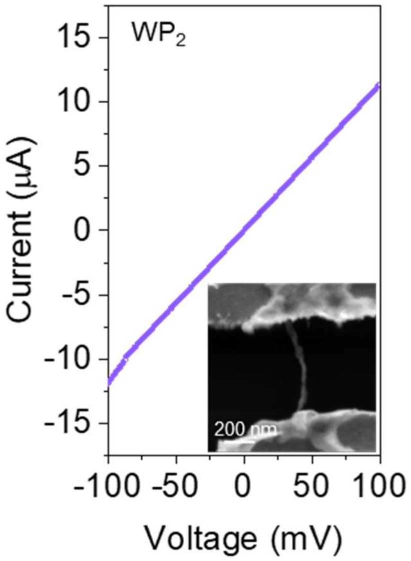

Supplementary Fig. 12. Source-drain current (I_{sd}) vs voltage (V_{sd}) for a 35.5 nm α-WP_2 nanowire.

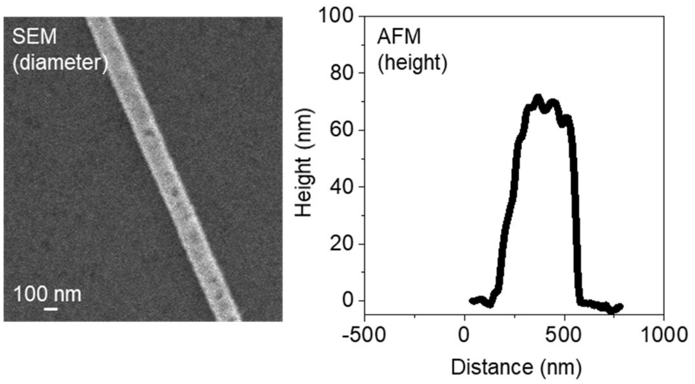

Supplementary Fig. 13. SEM and AFM line profiles for checking diameter and thickness.

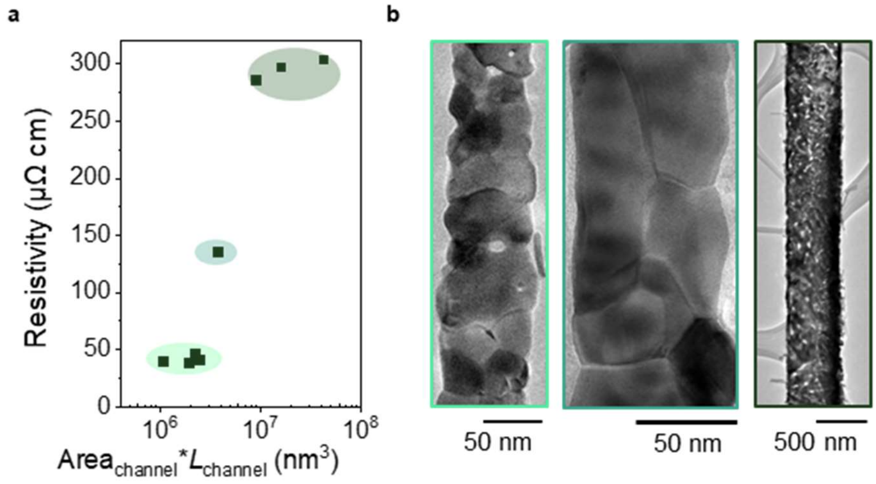

Supplementary Fig. 14. Correlation between the resistivity and grain structures for WP nanostructures with corresponding TEM images. **a**, Room temperature resistivity data of 1D-confined WP with varying cross-sectional area*channel length. **b**, TEM image of 1D-confined WP with varying volumes.

	100	010	001	110	101	011	Average from Wulff construction
WP	12.024	13.399	12.403	13.237	9.464	8.417	9.162
WP2	8.552	10.347	10.081	7.385	12.263	9.201	8.516

Supplementary Table 1. Calculated surface energy in eV nm^{-2} for WP and WP_2.

Material	r_{film} (10^{-16} Ωm^2)	r_{wire} (10^{-16} Ωm^2)
Cu	6.1	6.2
MoP	8.3	8.3
MoP_2	58.4	31.67
WP	9.9	11.1
WP_2	10.1	10.3

Supplementary Table 2. Resistivity scaling descriptors: r_{film} and $r_{wire.}$ For definition and computation details, see Suppl. Ref. 1.

	ρ_a (μΩ cm)	ρ_b (μΩ cm)	ρ_c (μΩ cm)
WP	19.5	16.9	42.3
WP_2	43.7	47.5	14.9
MoP	12.9	12.9	9.8
MoP_2	45.7	51.6	30.9

Supplementary Table 3. Calculated bulk resistivity values along each crystal axis.

Supplementary References

1. Kumar, S., Multunas, C., Defay, B., Gall, D., & Sundararaman, R. Ultralow electron-surface scattering in nanoscale metals leveraging Fermi-surface anisotropy. *Phys. Rev. Materials* **6**, 085002 (2022).
